# Potential Strategies to Control the Risk of Antifungal Resistance in Humans: A Comprehensive Review

**DOI:** 10.3390/antibiotics12030608

**Published:** 2023-03-18

**Authors:** Ali A. Rabaan, Tarek Sulaiman, Shamsah H. Al-Ahmed, Zainab A. Buhaliqah, Ali A. Buhaliqah, Buthina AlYuosof, Mubarak Alfaresi, Mona A. Al Fares, Sara Alwarthan, Mohammed S. Alkathlan, Reem S. Almaghrabi, Abdulmonem A. Abuzaid, Jaffar A. Altowaileb, Maha Al Ibrahim, Eman M. AlSalman, Fatimah Alsalman, Mohammad Alghounaim, Ahmed S. Bueid, Awad Al-Omari, Ranjan K. Mohapatra

**Affiliations:** 1 Molecular Diagnostic Laboratory, Johns Hopkins Aramco Healthcare, Dhahran 31311, Saudi Arabia; 2 College of Medicine, Alfaisal University, Riyadh 11533, Saudi Arabia; 3 Department of Public Health and Nutrition, The University of Haripur, Haripur 22610, Pakistan; 4 Infectious Diseases Section, Medical Specialties Department, King Fahad Medical City, Riyadh 12231, Saudi Arabia; 5 Specialty Paediatric Medicine, Qatif Central Hospital, Qatif 32654, Saudi Arabia; 6 Department of Family Medicine, Primary Healthcare Center, Dammam 32433, Saudi Arabia; 7 Directorate of Public Health, Dammam Network, Eastern Health Cluster, Dammam 31444, Saudi Arabia; 8 Department of Pathology and Laboratory Medicine, Zayed Military Hospital, Abu Dhabi 3740, United Arab Emirates; 9 Department of Pathology, College of Medicine, Mohammed Bin Rashid University of Medicine and Health Sciences, Dubai 505055, United Arab Emirates; 10 Department of Internal Medicine, King Abdulaziz University Hospital, Jeddah 21589, Saudi Arabia; 11 Department of Internal Medicine, College of Medicine, Imam Abdulrahman Bin Faisal University, Dammam 34212, Saudi Arabia; 12 Infectious Diseases Department, King Fahad Specialist Hospital, Buraydah 52382, Saudi Arabia; 13 Organ Transplant Center of Excellence, King Faisal Specialist Hospital and Research Center, Riyadh 11211, Saudi Arabia; 14 Medical Microbiology Department, Security Forces Hospital Programme, Dammam 32314, Saudi Arabia; 15 Microbiology Laboratory, Laboratory Department, Qatif Central Hospital, Qatif 32654, Saudi Arabia; 16 Department of Family Medicine, Primary Health Care Centers, Qatif Health Network, Qatif 31911, Saudi Arabia; 17 Department of Emergency Medicine, Oyun City Hospital, Al-Ahsa 36312, Saudi Arabia; 18 Department of Pediatrics, Amiri Hospital, Kuwait City 13041, Kuwait; 19 Microbiology Laboratory, King Faisal General Hospital, Al-Ahsa 31982, Saudi Arabia; 20 Research Center, Dr. Sulaiman Al Habib Medical Group, Riyadh 11372, Saudi Arabia; 21 Department of Chemistry, Government College of Engineering, Keonjhar 758002, India

**Keywords:** antifungal, antifungal resistance, fungal infections, fungal treatments

## Abstract

Fungal infections are becoming one of the main causes of morbidity and mortality in people with weakened immune systems. Mycoses are becoming more common, despite greater knowledge and better treatment methods, due to the regular emergence of resistance to the antifungal medications used in clinical settings. Antifungal therapy is the mainstay of patient management for acute and chronic mycoses. However, the limited availability of antifungal drug classes limits the range of available treatments. Additionally, several drawbacks to treating mycoses include unfavourable side effects, a limited activity spectrum, a paucity of targets, and fungal resistance, all of which continue to be significant issues in developing antifungal drugs. The emergence of antifungal drug resistance has eliminated accessible drug classes as treatment choices, which significantly compromises the clinical management of fungal illnesses. In some situations, the emergence of strains resistant to many antifungal medications is a major concern. Although new medications have been developed to address this issue, antifungal drug resistance has grown more pronounced, particularly in patients who need long-term care or are undergoing antifungal prophylaxis. Moreover, the mechanisms that cause resistance must be well understood, including modifications in drug target affinities and abundances, along with biofilms and efflux pumps that diminish intracellular drug levels, to find novel antifungal drugs and drug targets. In this review, different classes of antifungal agents, and their resistance mechanisms, have been discussed. The latter part of the review focuses on the strategies by which we can overcome this serious issue of antifungal resistance in humans.

## 1. Introduction

The human population has been plagued by various infectious diseases for a long time and they remain one of the leading causes of death [1]. Fungal infections are not considered a severe health issue, even though they affect over a billion people. Approximately 1 million individuals have invasive fungal infections (*Aspergillus*, *Candida*, *Cryptococcus*, and *Pneumocystis*) worldwide, resulting in the death of 1.7 million people annually [2,3]. The WHO has developed a priority list of fungi based on the severity of infection, the WHO FFPL. This is the first global effort to systematically classify fungal pathogens. Nineteen fungi were categorized into three priority groups (critical, high, and medium) in this list considering their unfulfilled requirements in research and development and their significant role in public health [4]. Almost every person is affected by a superficial fungal infection, which is easy to cure. However, individual infection by a specific invasive fungal pathogen can be fatal due to a lack of definitive diagnosis and treatment [5]. For instance, recent studies estimated ~300,000 cases of invasive *Aspergillosis*, 750,000 cases of *Candidiasis*, and more than 900,000 cases of *Mucormycosis* infection annually [6,7]. Additionally, the emergence of antifungal drug resistance in susceptible pathogens (the ubiquitous mould *Aspergillus fumigatus*) and novel fungal species resistance to multiple antifungal drugs (for example, the yeast *Candida auris*) are alarming critical concerns to human health worldwide [8,9,10,11]. The inaccessibility of efficient therapeutics against such fungal infections and the gain of resistance in fungi against antifungal agents is, therefore, contributing a hindrance to eradicating fungal infection while increasing the mortality rate [12]. In addition, an increasing number of diseased populations, such as older people and immunodeficient or seriously ill patients, such as AIDS, cancer, diabetic patients, and transplantation patients, are vulnerable to fungal disease, which provides a perfect niche for infection [13]. Additionally, fungal infections were connected with other bacterial and viral infections, including severe acute respiratory syndrome coronavirus 2 (SARS-CoV-2); immunocompromised patients thereof are prone to invasive fungal infections [14]. For instance, the most unambiguous indication of co-infection was the appearance of mucormycosis, a fungal infection caused by Mucorales, in South Asian nations, particularly in India, following the first wave of the coronavirus diseases 19 (COVID-19) caused by SARS-CoV-2. [15,16]. However, it appears that severe immunocompromised hosts (such as those who have undergone organ transplantation, cancer, autoimmune disorders, immune system functional limitations, or neutropenia), hyperglycaemia or poorly controlled diabetes mellitus, and immunocompetence in post-traumatic cases are the leading causes of this infection [15].

**Figure 1:**
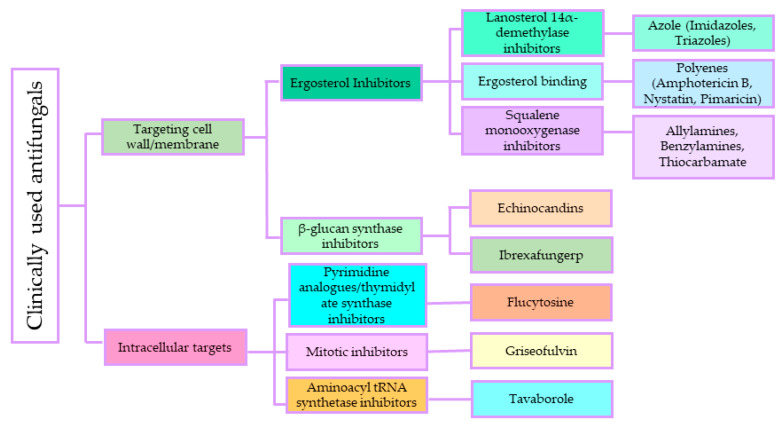
Flow chart depicting classification of antifungal drugs concerning the mode of action as well as names of drugs used in the management of fungal infections in humans.

Among the known fungal pathogens, drug-resistant pathogens in invasive fungal infections are responsible for causing more than 50% of the mortality rate; therefore, it is essential to understand the mechanism of drug resistance and the drawbacks of the currently available antifungal treatments for the clinical management of fungal infections [5]. Recent studies established that pathogenic fungi acquired various adaptive mechanisms to achieve antifungal drug resistance, such as alteration of the target drug site, target overexpression, upregulation of the multidrug transporters, biofilm formation, cell permeability, and stress response [17]. For instance, fungi gained primary resistance against the chemical action of antifungal drugs using intrinsic resistance parameters, including biofilm formation and cell wall impermeability. After continuous exposure to antifungal agents, fungi acquired secondary mechanisms to adopt resistance, termed acquired resistance, via genetic alternations to modify the target binding site or overexpression of the targeted proteins, or stress response and stress response/epigenetic pathways alternation [18,19,20,21]. Additionally, fungi can also gain antifungal drug resistance via random mutations induced by the overuse of antifungal functional groups, such as azole and echinocandins [22]. Therefore, a systematic treatment for fungal infections is mainly based on four classes of antifungal drugs, i.e., polyenes, azoles, echinocandins, and antimetabolite agents (Figure 1) [19]. For example, heptaene amphotericin B is a class of polygene which binds to ergosterol, a significant component of the fungal cell membrane. It creates pores in the cell membrane to cause leakage of intracellular ions. Such drugs have been documented for the fungicidal or fungistatic activity against *Aspergillus fumigatus*, *A. flavus*, and *Candida* genera [23]. Likewise, azole drugs interfere with the ergosterol biosynthesis by inhibiting the ergosterol biosynthesis of the 14α-lanosterol demethylase in fungi and are reported with potential activity against yeast and *Aspergillus* species [24]. Additionally, echinocandins (containing caspofungin, micafungin, and anidulafungin drugs) are known to inhibit the synthesis of β-d-glucans in fungi, which are essentially required for the formation of the fungal cell wall [25]. Similarly, flucytosine, an antimetabolite agent that inhibits fungal growth by alternating in fungal DNA and protein synthesis, is generally administered in conjunction with amphotericin B to treat refractory *Candida* infections and *Cryptococcal meningitis* infections [26]. Pathogenic fungi respond to these available antifungal agents effectively. Still, with prolonged usage of these drugs and due to some external factors, the pathogen attains resistance, resulting in no improvement in the infected individual even after treatment [27]. Other factors, such as a defective host immune system, poor antifungal activity, and specific fungal characteristics, such as antifungal tolerance and resistance, were also elucidated as critical factors in promoting antifungal drug resistance or the failure of antifungal treatment [28]. It is also important to mention that over-using antifungal drugs increases the chance of opportunistic pathogens attaining resistance [29]. So, it is essential to find specific and practical strategies to treat drug-resistant fungi by analysing the molecular mechanism leading to drug resistance, which can help identify new potential targets for developing new antifungal drugs [30]. Hence, the present review gives a brief understanding of the development of drug resistance in fungi against the commonly used antifungal agents and potential strategies adopted to overcome antifungal resistance, mainly in humans.

## 2. Methodology

### 2.1. Literature Screening

In this study, PubMed (https://pubmed.ncbi.nlm.nih.gov/, accessed on 20 June 2022) search engine was selected as they use the MEDLINE database to find relevant papers on life sciences and biomedicines. This search engine helps to screen the articles based on various criteria such as year of publication, type of article, text available, and other additional filters to make the screening of articles easier and accurate. Keywords ‘Antifungal’, ‘Antifungal resistance’, ‘Fungal infections’, ‘Fungal treatments’, and ‘Antifungal agents’ were used to search and collect the relevant literature from this database. Additionally, Clinical Trials (https://clinicaltrials.gov/, accessed on 20 June 2022) database was also considered as the secondary database to find the relevant information. Initially, the first search on the mentioned databases was performed on 26 June 2022, and relevant articles, both research and review papers, published between 2001 and 2022 were collected. Later, a second literature collection was conducted on 1 July 2022, to update (1 October 2022), and again, the most relevant publications were downloaded and considered in the present review. Following that, the collected literature was screened using the multiple keywords mentioned above to assemble the most pertinent publications.

### 2.2. Exclusion of Articles

Conclusively, the adopted methodology for the literature screening resulted in a collection of 7500 hits. This set of articles was further refined and articles containing information about the non-antifungal drugs (1500 articles), such as antiparasitic drugs and diseases, were excluded. Furthermore, from the available 6000 hits, another 4000 articles on antifungal agents related to plants and animals (2500 papers) were also removed from the set of selected papers. A set of articles containing incomplete information regarding antifungal resistance and published in languages other than English (1000 publications) were also excluded as they did not fulfil the selection criteria. Additionally, around 500 articles were found to be duplicates. 

### 2.3. Collection of Data from Relevant Articles

**Figure 2:**
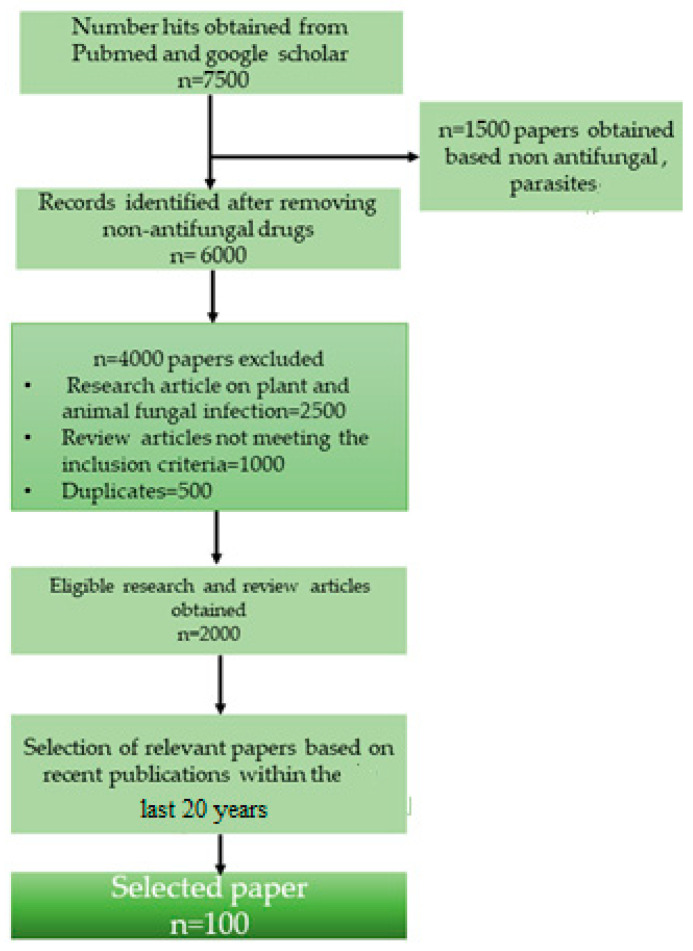
Flow chart exhibiting selection criteria adopted for selecting research publications used in the design and compilation of the present review.

From the remaining 2000 eligible research and review papers, only 100 relevant papers focusing on the inclusion criteria, such as evaluating antifungal resistance using in vitro and in vivo methods, various mechanisms of antifungal drug resistance shown by prominent fungal pathogens, and strategies used for the prevention of antifungal resistance, were sorted for the present systematic review. As the current paper concentrates on antifungal resistance in humans, 100 articles within the last 20 years were thus further sorted for a new collection that discussed antifungal resistance or antifungal medication in humans. To provide a comprehensive understanding of antifungal drug resistance, 60 articles were consulted from these 100 selected publications that describe the categorisation of antifungal medications, their mechanisms of action, the development of antifungal resistance, and strategies to overcome antifungal resistance in humans for a detailed discussion. In addition, some relevant articles were cited and discussed during the article revision. A systematic flow representing the adopted methodology for selecting and refining the literature is given in Figure 2. 

## 3. Results and Discussion

The overuse of existing antifungal medications in recent years has led to the evolution of antifungal-resistant fungi or the emergence of novel fungal species with solid antifungal tolerance [31]. As a result of its impact on human health, there is now a great deal of interest in understanding antifungal resistance from various viewpoints. The existing antifungal therapeutic agents are affected by other factors such as drug–drug interaction, toxicity, and limitation in routes of drug administration [32]. In the last two decades, no new antifungal classes have been available, and only a few new antifungal drugs of the existing class of drugs are being approved for treatment. Identification of novel antifungal pathways and drug targets is utilised for developing potential antifungal drugs with a novel mode of action or for a new formulation of existing antifungals [33,34]. A brief insight into the existing antifungal drugs and their resistance mechanisms will be helpful in the formulation of new antifungal agents. 

### 3.1. Antifungal Agents and Their Mode of Action

Antifungal agents can be divided into nonspecific and specific agents based on the mode of action. Nonspecific antifungals mainly include disinfectants, antiseptics, and essential oils that can be applied to treat skin and mucous antifungal infections. Specific antifungals, also known as antimycotic drugs, have a particular mechanism of action [35]. For example, polyenes, antimetabolites agents, azoles, and echinocandins are the four classes of specific antifungal drugs approved by the Food and Drug Administration (FDA) for treating invasive fungal infections [36]. Only certain antifungals are taken into consideration in the current investigation because they are commonly used to treat invasive fungal infections. 

#### 3.1.1. Polyenes

The first antifungal polyene—fungicidin, later called nystatin—was discovered in 1949. More than 200 polyenes as fungal-specific antibiotics have been discovered and used to treat fungal infections in humans [36,37]. These antifungal agents show fungicidal activity against numerous species of *Aspergillus*, *Candida*, and *Cryptococcus* genera [17]. Generally, polyenes possess a cyclic heptaene or polyketide core macrolactone ring (20–40 carbon atoms including 3–8 conjugated double bonds) structure and are produced by the Gram-positive bacterium *Streptomyces nodosus*. Based on the conjugated double bonds, polyenes are characterised as trienes, tetraenes, pentaenes, hexaenes, heptaenes, etc. [38,39]. Approximately six polyenes were identified that could be used for antifungal therapy: amphotericin B, nystatin, natamycin (also called pimaricin), candicidin, trichomycin, and methyl partricin [37]. Amphotericin B (amphotericin B deoxycholate) is the most widely used and was first approved by the FDA to treat invasive fungal infections but, due to low therapeutic index and high nephrotoxicity, the usage of this drug has been limited [33,36]. To improve drug quality and reduce drug toxicity, liposomal amphotericin B was introduced, where the amphotericin B is induced into a small, unilamellar vesicle made up of a liposomal formulation. The liposomal amphotericin B is much safer and less toxic than the conventional amphotericin B and is used for the treatment of various fungal infections [40].

**Figure 3:**
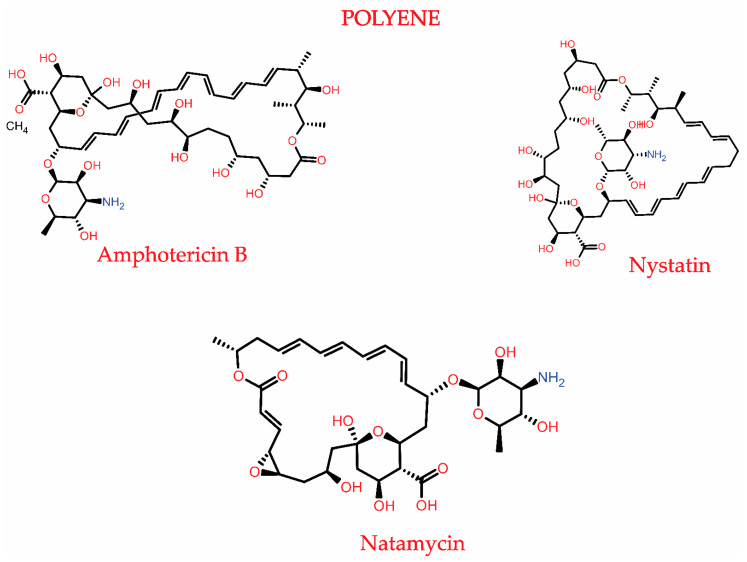
Two-dimensional structural formula for the most common drugs under the polyene antifungal group. All the 2D structures were plotted using the academic version of Maestro Schrödinger suite 2022 (Schrödinger Release 2022-3: Maestro, Schrödinger, LLC, New York, NY, USA, 2021).

These antifungal agents exhibit two different modes of action against the fungi, i.e., (i) the polyenes incorporate into the fungal lipid bilayer and bind to the ergosterol molecule. This results in pore formation in the fungal cell wall and causes leakage of essential ions (K^+^, Mg^2+,^ Ca^2+^, and Cl^−^) and energy molecules (glucose), leading to the death of fungal cells; (ii) polyenes instigate reactive oxygen species (ROS) production and accumulation in fungi, which causes substantial damage to the fungal protein, mitochondria, cell membrane, and DNA [41]. Another mode of action of polyene is the extraction or ergosterol adsorption from the membrane leading to membrane destabilisation and membrane protein function disturbance [42] (Table 1, Figure 3).

#### 3.1.2. Antimetabolite Agents (5-Fluorocytosine)

**Figure 4:**
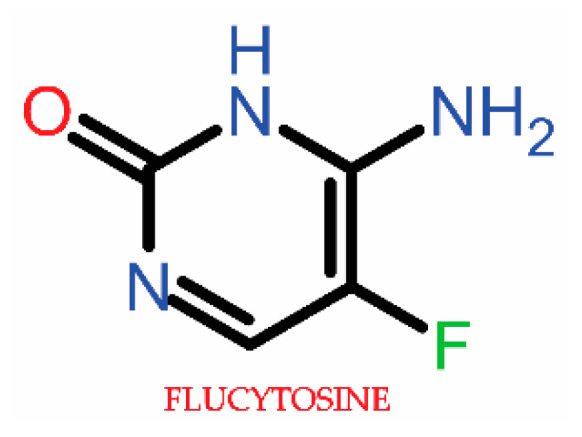
Two-dimensional structure of flucytosine used as an antimetabolite agent against invasive fungal infections.

Antimetabolites are structurally characterized as analogues of essential metabolites but cannot be consumed by the human body [43]. The common classes of antimetabolites include purine antagonists (6-mercaptopurine) [44] and pyrimidine antagonists (5-fluorouracil) [45]. Some natural products, such as vinca alkaloids and taxol, have been reported as antimetabolites in disease management [46,47]. Some antimetabolites are also available for use against invasive fungal infections in humans. The best example is 5-fluorocytosine, a synthetic analogue of cytosine, which was first synthesized in 1957 as an antitumor drug and later approved by the FDA as an antifungal drug for humans in 1968 (Figure 4). Flucytosine effectively treats fungal infections caused by *Cryptococcus neoformans*, *Candida* spp., and *Cryptococcal meningitis* [48,49,50]. It is also used with amphotericin B to treat systemic mycoses, and this association also reduces the rate of nephrotoxicity in patients compared to treatment of amphotericin B alone [51,52]. Additionally, the combination of these drugs has a higher efficacy rate [53]. Typically, when the drug enters the fungus, the drug gets activated into 5-fluorouracil(5-FU) by the fungal cytosine deaminase enzyme and impedes DNA and RNA synthesis via intracytoplasmic conversion. For instance, 5-fluorouracil (5-FU) is converted into 5-fluorouridine monophosphate (5FUMP) with the help of uracil phosphoribosyltransferase (FUR1) and then 5-fluorouridine triphosphate to alter the RNA synthesis as well as protein synthesis [54,55,56]. Moreover, fluoro-deoxyuridylic acid, the modified form of 5-fluorouracil (5-FU), also inhibits DNA synthesis and causes DNA damage by the inhibition of thymidylate synthase [55] (Table 1).

#### 3.1.3. Azole

**Figure 5:**
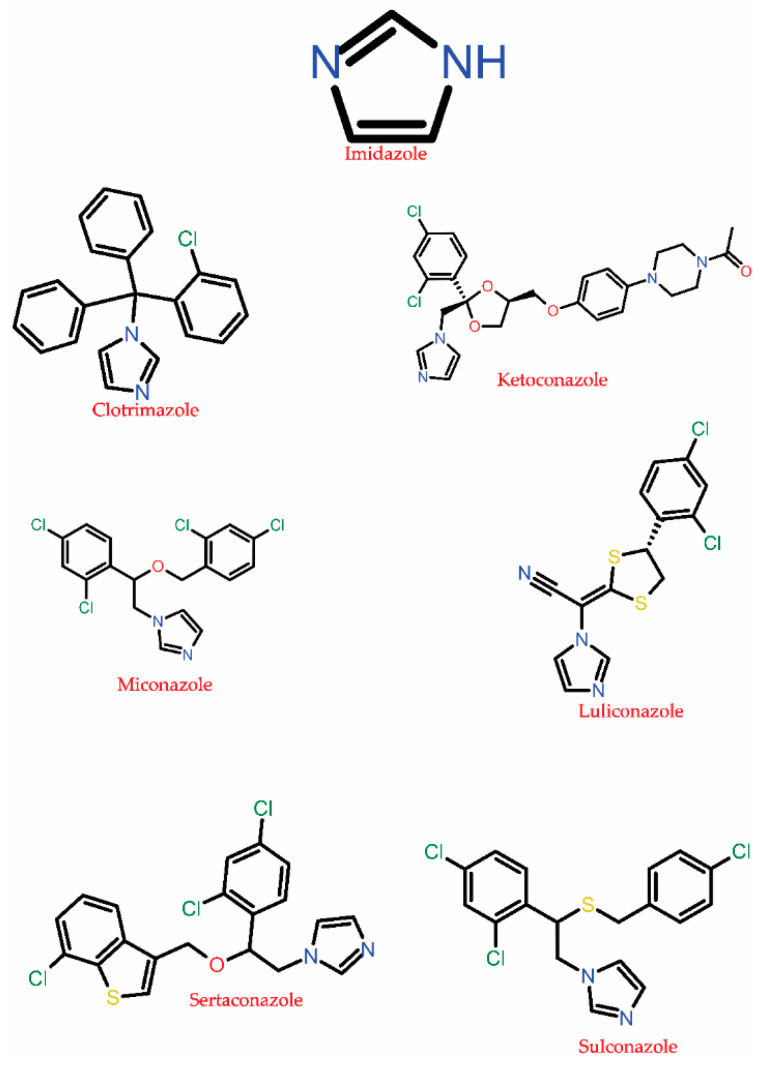
Two-dimensional structure of drugs under the imidazole group.

**Figure 6:**
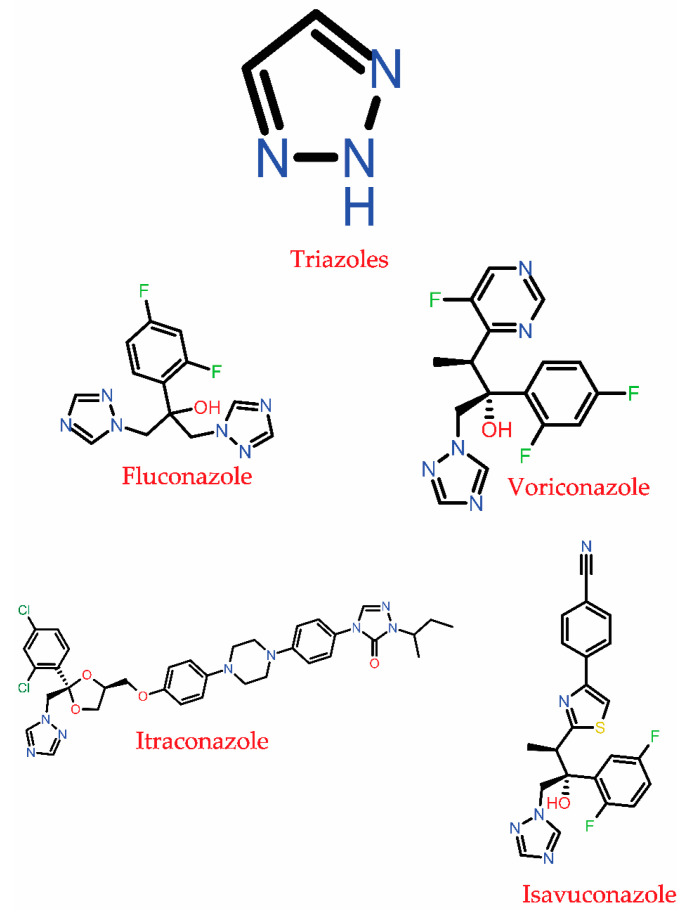
Two-dimensional structure of drugs under triazole group.

**Figure 7:**
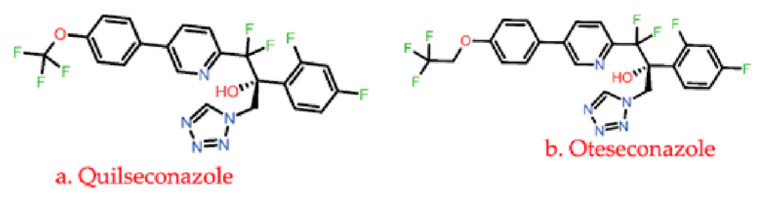
Two-dimensional structure of drugs under tetrazole group.

Azoles, a broad-spectrum class of antifungals, were discovered in 1944 and approved for human use in the 1950s [36]. Based on the number present in the aromatic ring of azoles, they are classified into three groups: imidazoles, triazoles, and tetrazoles. Imidazoles have two nitrogen atoms in the azole ring, and triazoles have three nitrogen atoms in the azole ring [57]. Miconazole, clotrimazole, econazole, ketoconazole, tioconazole, sulconazole, serconazole, and luliconazole represents the imidazole-based azole drugs and terconazole, fluconazole, isavuconazole (isavcuconazonium—prodrug of isavuconazole), itraconazole, voriconazole, posaconazole, eficonazole, and albaconazole represents the triazole group of drugs [58]. Tetrazole, a recently developed compound under the azole group, shows broad spectrum activity against fungal species, but its usage is limited as most of the drugs are under trial stage and need to be approved by worldwide agencies [59,60]. Tetrazoles, which include one carbon and four nitrogen atoms, are a subclass of doubly unsaturated aromatic heterocycles with a five-membered ring. In nature, they do not exist [61]. Modified tetrazoles named quilseconazole (VT-1129) and oteseconazole (VT-1161) are the latest developed inhibitors of lanosterol 14α-demethylase encoded by the Cyp51 gene [54,62]. They are designed to overcome the problem of drug–drug interaction, a significant limitation of the azole class of antifungal drugs. These are developed by Mycovia Pharmaceuticals (previously known as Viamet Pharmaceuticals), Inc. (Durham, NC, USA) by replacing the triazole metal binding group with a tetrazole. This modification results in the development of more specific compounds in the inhibition of the *Cyp51* gene and shows minor drug–drug interaction [63,64]. The FDA approved quilseconazole as an orphan drug for the treatment of cryptococcal meningitis, and oteseconazole is approved for the treatment of recurrent vulvovaginal candidiasis (RVVC) [54,65,66]. Quilseconazole and oteseconazole show antifungal activity against *Candida* and *Cryptococcus* species [67]. Drugs under the azole group inhibit the 14α-lanosterol demethylase enzymes, causing a disturbance in ergosterol biosynthesis, a significant cell membrane component. As a result, the fungal cells experience depletion of ergosterol and accumulation of toxic 14-methylated sterols, leading to cell lysis and death [68]. Azoles displays fungicidal and fungistatic activity against fungi from genera *Candida*, *Cryptococcus*, *Coccidioides*, *Aspergillus*, yeasts, and moulds. Voriconazole, isavuconazole, and itraconazole are the most preferred drugs for the treatment of invasive aspergillosis and fluconazole is one of the most safest and effective drugs used for the treatment of candida endophthalmitis [69,70,71,72,73] (Table 1) (Figure 5, Figure 6 and Figure 7). 

#### 3.1.4. Echinocandins

**Figure 8:**
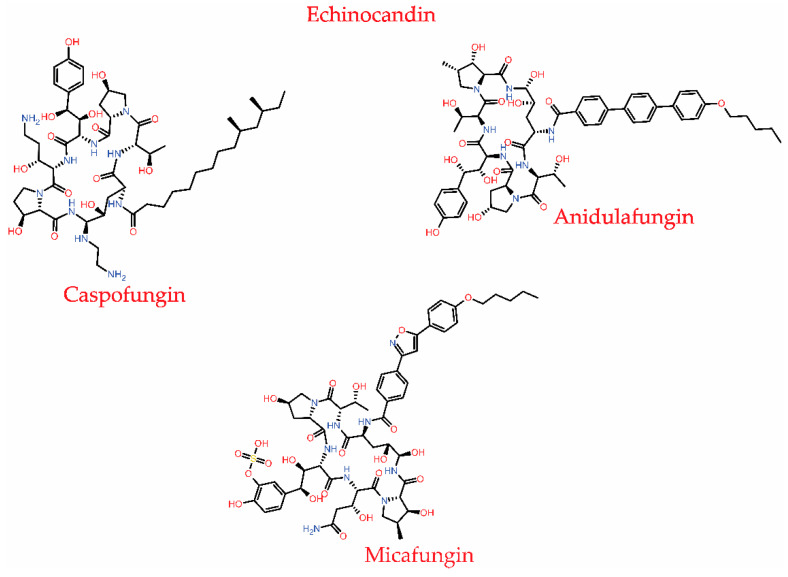
Two-dimensional structure of drugs under the echinocandin antifungal group.

Echinocandins, cyclic amphiphilic peptides with long lipophilic side chains, are the most recent class of antifungal drugs for the treatment of fungal infections; for example, caspofungin, micafungin, and anidulafungin belong to echinocandins, which are approved by the FDA and the European medicine agency for intravenous administration [74]. Echinocandins act as non-competitive inhibitors of β-1,3 glucan synthase, an essential enzyme complex responsible for cell wall synthesis in fungi [75]. This enzymatic disruption by echinocandins results in a leaky fungal cytoplasm and alters osmotic pressure, followed by fungal cell lysis, demonstrating fungicidal activity [76,77,78,79]. This fungicidal activity of echinocandins has been observed in the fungal species of *Candida* and *Saccharomyces*. Notably, due to the absence of cell walls in mammalian cells, it is less toxic and has significantly less drug–drug interaction than other antifungal drugs, but they show low penetration into brain and CSF, due to which they are not preferred for the treatment of fungal infection in these tissues [76,80]. Echinocandins also demonstrated fungicidal activity against *Candida* spp. and fungistatic activity via inducing structural alterations in the fungus against *Aspergillus* spp. [81,82,83,84] (Table 1) (Figure 8). 

### 3.2. Resistance to Antifungal Agents

Antifungal resistance is an emerging worldwide issue due to new resistant variants of the existing fungal pathogens, for example, *Aspergillus fumigatus* and *Candida auris* [9,85]. To reduce toxicity in a host cell, the antifungal drug must act towards specific targets, which are not conserved between the fungi and the human host [17]. To overcome the issue of drug resistance, it is essential to understand the mechanism of drug resistance to antifungal drugs. Intrinsic and acquired resistance are two significant consequences of long-term treatment and a high range of prophylaxis [54]. The following sections briefly describe the mode of resistance gain in the fungi against four different classes of specific antifungal drugs. 

#### 3.2.1. Polyene Resistance

Mechanisms of resistance to polyenes involve modification in the fungi membrane sterols, instigating antioxidant mechanisms to halt the damage caused by oxidative stress, and alternations in the ergosterol biosynthetic genes. Notably, twenty-five different genes are deciphered to play a significant role in regulating ergosterol biosynthesis (Table 1). For example, alternations (*ERG3*, *ERG5*, and *ERG11*), deletion (*ERG11*), and mutation (*ERG3, ERG5*, and *ERG11*) in the genes encoding respective enzymes were found to decrease the efficacy of amphotericin B in the treatment of infection caused by *Candida* species [86]. Mutation in the *MEC3*, a gene responsible for DNA damage homeostasis, also contributes to the high MIC of polyene in *C. auris* [87]. Moreover, overexpression of the molecular chaperones, such as the heat shock proteins (Hsp90 and Hsp70) family, further contributes to the development of intrinsic resistance in Aspergillus terreus against amphotericin B [88,89]. Alteration in the fungal cell wall composition, significantly increasing the 1,3-α-glucan and 1,3-β-glucan fraction, also contributes to amphotericin B resistance. This condition was seen in the amphotericin-B-resistant strains of *C. tropicalis*. The increase in the 1,3-β-glucan is seen to be responsible for strengthening the immune response and survival of resistant strains [90]. 

#### 3.2.2. Antimetabolite Agent Resistance

Flucytosine drugs make the pathogen easily resistant to the drug when used as monotherapy [91]. This is mainly used in combination therapy, along with amphotericin B and triazoles. It was found that some *Candida* spp. and *Saccharomyces cerevisiae* were resistant to this due to alternation in the *FCY2*, *FCY1*, and *FUR1* genes (Table 1), which are required for the uptake and conversion of flucytosine [92]. Another resistance mechanism reported is the upregulation of pyrimidines in de novo synthesis, leading to functional disturbance in uridine monophosphate pyrophosphorylase [91]. 

#### 3.2.3. Azole Resistance

The most common mechanism in azole resistance is the alternation or overexpression of the *ERG11*, *Cyp51A*, and *Cyp51B* genes, which encode the lanosterol 14-alpha-demethylase enzyme belonging to the cytochrome P450 family [93]. For instance, overexpression of *ERG11* is due to the functional mutation in the Upc2 transcriptional activator in *C. albicans* [29]. Similarly, *A. fumigatus* becomes resistant to azole when alternation occurs in the *Srb* transcriptional factor, *HapE* modification, *Cyp51B* overexpression, and biofilm formation [18,94,95]. Upregulation of the *ABC* (adenosine triphosphate binding) cassette also causes acquired resistance to azole in *C. albicans* and *C. glabrata* [29]. The mutation of the *RAD50* gene in the double-strand break repair (DSBR) and the *MSH2* and *PMS1* genes of mismatch repair (MMR) of the DNA repair pathway are responsible for inducing fluconazole resistance in fungi. Additionally, cerebellar degeneration-related protein 1 and 2 (CDR1, CDR2) and SNQ2 are the transporters responsible for resistance to azole in fungi. For instance, when the SNQ2 transporter is removed from these groups, it was found to restore azole susceptibility, making it an essential factor in azole resistance [96]. Additionally, azole resistance was observed in *Candida dubliniensis*, a pathogenic yeast with phenotypic characteristics similar to *Candida albicans*, which mainly infects patients with HIV/AIDS. *C. parapsilosis*, *C. tropicalis*, and some non-albicans species were also found to be resistant against azoles and typically gained azole resistance by drug efflux, drug target modification, and alternation in the ergosterol biosynthesis [97,98] (Table 1). 

#### 3.2.4. Echinocandin Resistance

The echinocandin resistance mainly occurs due to the amino acid substitution or point mutation in the *FKS1 and FKS2* subunits in glucan synthase [99]. In *C. albicans*, the change occurs in Ser^641^ and Ser^645^ and in *C. glabrata*, it is seen in Ser^629^, Phe^659^, and Ser^663^; both these changes lead to resistance [100]. Resistance mediated by *FKS2* is only seen in *C. glabrata* and can be reversed using the calcineurin inhibitor tacrolimus [18,29]. In *A. fumigatus*, in vitro resistance of echinocandin was found when induced with the substitution of Ser^678^ in *FSK1* [94] (Table 1).

**Table 1:** List of all four groups of antifungal agents and their resistance mechanism.

Antifungal Agents	Mode of Action	Example of Antifungal Resistant Species	Resistance Mechanism	Reference
**Polyenes** **Amphotericin B** **Nystatin** **Natamycin**	○ Cell membrane disruption by binding to ergosterol. ○ Excessive production and accumulation of reactive oxygen species (ROS) causing damage to the cell membrane, DNA, fungal protein, and mitochondria	*Candida species*, and *Aspergillus terreus*	Alternation (*ERG3*, *ERG5*, and *ERG11*), deletion (*ERG11*), and mutation (*ERG3*, *ERG5*, and *ERG11*) genes encoding for C-5 sterol desaturase, C-22 sterol desaturase, and sterol 14-demethylase are responsible for the regulation of ergosterol biosynthesis.	[36,41,93]
**Antimetabolite agent** **Flucytosine**	Inhibits RNA and protein synthesis by binding 5-fluorouracil(5-FU) to the RNA strand.	*Candida* spp.	Alternation in the *FCY2*, *FCY1*, and *FUR1* genes that are responsible for the uptake and conversion of flucytosine.	[54,55,92]
**Azole** **Imidazole** ○ **Miconazole** ○ **Clotrimazole** ○ **Econazole** ○ **Ketoconazole** ○ **Tioconazole** ○ **Sulconazole** ○ **Sertaconazole** ○ **Luliconazole** ○ **Tinidazole** ○ **Enilconazole** ○ **Parconazole** ○ **Eberconazole** ○ **Lanoconazole** ○ **Fenticonazole** ○ **Bifonazole** ○ **Sulconazole** ○ **Lombazole** ○ **Sertaconazole** ○ **Oxiconazole** ○ **Butaconazole** ○ **Isoconazole** ○ **Flutrimazole** ○ **Ornidazole** ○ **Metronizadole** **Triazoles** ○ **Itraconazole** ○ **Fluconazole** ○ **Voriconazole** ○ **Posaconazole** ○ **Isavuconazole** ○ **Fosravuconazole** ○ **Albaconzole** ○ **Letrozole** ○ **Anastrozole** **Tetrazole** ○ **Quilseconazole (VT-1129)** ○ **Oteseconazole (VT-1161)**	Inhibition of fungal lanosterol 14α-demethylase cytochrome P450 51, P450 3A4, P450 2D6, P450 2C8, P450 2A6, P450 2E1 P450 2C9, etc., that disrupts the ergosterol biosynthesis leading to cell death and lysis. Some of these drugs also targets DNA, bile salt export pump, P-glycoprotein 1, Hydroxycarboxylic acid receptor 2, oxygen-insensitive NADPH nitroreductase.	*C. albicans*, *C. glabrata*, *C. dubliniensis* *A. fumigatus*, yeast, *Trichophyton rubrum*	Alternation and overexpression of *ERG11*, *Cyp51A*, and *Cyp51B* encodes for lanosterol 14-alpha-demethylase enzyme.	[65,67,70,71,93,101,102]
**Echinocandin** **Caspofungin** **Micafungin** **Anidulafungin**	Inhibits β-1,3 glucan synthase, an essential enzyme complex responsible for cell wall synthesis in fungi.	*C. glabrata* and *A. fumigatus*	Amino acid substitution or point mutation in the *FKS1* and *FKS2* genes present in the glucan synthase	[75,99]

#### 3.2.5. Other Mechanisms Involved in Drug Resistance

(a) Biofilm formation

Biofilms are a surface-associated microbial communities attached by a self-made extracellular matrix [103]. The biofilm formation helps the organism withstand antimicrobial agents’ attack and survive severe environmental conditions [104]. In the case of fungal pathogens, such as *C. albicans*, biofilm formation is involved in the pathogenicity and death of patients [105,106]. Researchers found that *C. auris* adheres to a polymeric surface that further develops into a biofilm and can resist various antifungal agents, especially caspofungin, which are very effective against *Candida* biofilms [107]. Fungal biofilms also show resistance to echinocandin and azole antifungal drugs [108,109].

(b) Modification of drug targets

The drug targets are modified by specific genetic changes that lead to a reduction in the affinity of drugs. For example, modification of echinocandin drug targets confers drug resistance [110]. Mutation in the *FKS* gene that encodes β-D-glucan synthase leads to substituting amino acids that result in the target site modification [111,112]. The drug affinity becomes reduced by several folds due to the change of targets. The mutation in the ERG11 also results in amino acid substitutions that are responsible for azole resistance, and this further decreases the affinity of the azoles to the target site [113,114].

(c) Efflux pump

Fungal efflux is responsible for regulating the environment and removing toxic substances, including antifungal agents. The overexpression of various drug transporters leads to the efflux of the drug and also prevents the accumulation of antifungal medications in the cytosol [115]. Two major efflux pumps involved in drug resistance are the ATP-binding cassette (ABC) superfamily and the major facilitator superfamily (MFS). Among azole-resistant (AR) clinical isolates of *C. albicans*, CDR1 and CDR2 are two promiscuous ABC proteins that are upregulated [116,117].

### 3.3. Strategies to Overcome Resistance

A deeper understanding of antifungal resistance is important for developing effective counterstrategies to overcome it. With the emergence of antifungal resistance, the chances of survival of opportunistic pathogens have also increased, so it is important to find various practical approaches to eradicate drug resistance issues in pathogenic fungi. 

#### 3.3.1. Development of a New Antifungal Drug

Developing new antifungal drugs is one of the most essential strategies to reduce the risk of antifungal resistance. Several drugs have been identified and synthesized by researchers, which are currently in the preclinical and clinical trial stages. Some of the compounds have similar properties but may have better efficacy than the existing compound and some of the compounds may have a new mechanism of action.

(a) SUBA-itraconazole and VT-1598

The super bioavailable itraconazole (SUBA-ITC) is approved by the FDA and used as a first-line antifungal agent for the treatment of allergic bronchopulmonary aspergillosis (ABPA) and other fungal infections in children [118]. This antifungal formulation was developed by Mayne Pharma L.td., mainly to enhance the bioavailability of itraconazole. It also ensures the bioavailability by solid dispersion in a pH-dependent matrix and interferes with the cytochrome P450 activity to reduces the rate of ergosterol synthesis. This antifungal formulation also showed broad-spectrum activity against *Blastomycosis* sp., *Histoplasmosis* sp., and *Aspergillosis* sp. [54,119]. 

**Figure 9:**
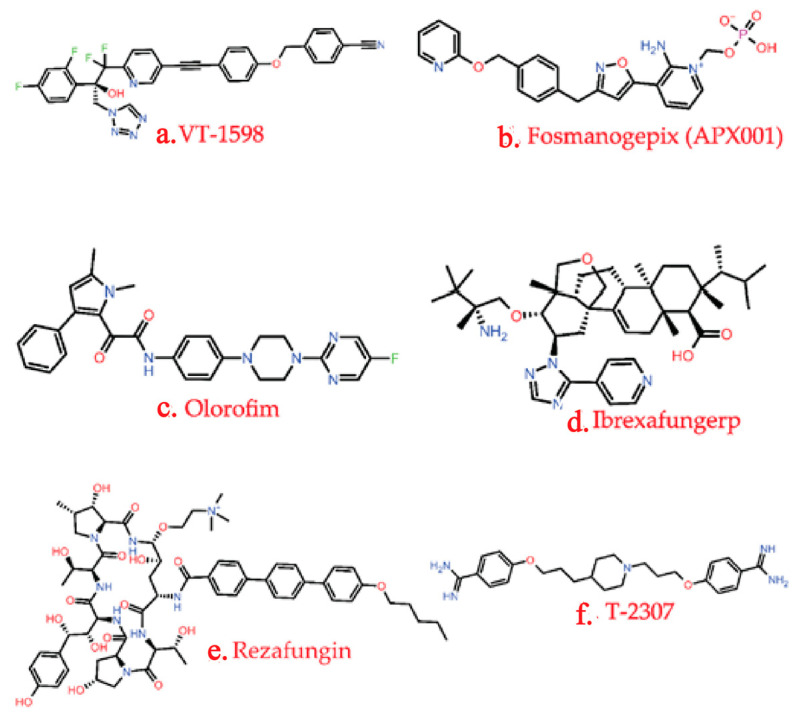
Two-dimensional structure of new antifungal agents developed.

Mycovia Pharmaceuticals has created VT-1598, the most recent tetrazole inhibitor of lanosterol 14-demethylase encoded by the *Cyp51* gene. VT-1598, which is still in Phase I of the clinical study, is effective against moulds, including Aspergillus species, Coccidioides species, and *Rhizopus arrhizus* [102,120] (Table 2) (Figure 9a).

(b) Rezafungin and Ibrexafungerp

Rezafungin, developed by Cidara Therapeutics, belongs to the class of echinocandins that inhibits 1,3-β-D-glucan synthesis and has been granted orphan drug designation by the FDA for the treatment of vulvovaginal candidiasis. It has been reported effective against *Candida* sp., *Aspergillus* sp., and *Pneumocystis* sp. [119,121]. Ibrexafungerp, an FDA-approved antifungal triterpenoid, was developed by SCYNEXIS Inc. and mainly inhibits 1,3-β-D-glucan synthesis. Both rezafungin and ibrexafungerp have low toxicity, are highly bioavailable, and significantly inactivate the *Candida* sp., including *C. glabrata* and *C. auris* [122] (Table 2) (Figure 9d,e).

(c) Olorofim

Another drug in development is olorofim, which comes under a new class of antifungal drugs called orotomides and is in the Phase II stage of a clinical trial. This class has a novel mode of action, where it inhibits the dihydroorotate dehydrogenase enzyme, an important enzyme in pyrimidine biosynthesis in fungi, to disturb the nucleic acid and cell wall synthesis. This class of antifungal has been reported with potential efficiency against *Aspergillus* sp. and *Scedosporium* sp. [32,123] (Table 2) (Figure 9c).

(d) Amphotericin B Cochleate (CAMB)

The amphotericin B cochleate (CAMB), an oral formulation of amphotericin B, is a new drug derived from the polyene class and is under the Phase II stage of clinical trials, developed by Matinas BioPharma. CAMBs are formulated using phosphatidylserine along with phospholipid-calcium and are stable against degradation in the gastrointestinal tract. This formulation was successfully reported to treat *C. albicans* infection in a murine model [124] (Table 2).

(e) MGCD290

MGCD290 is an orally administered drug that inhibits the Hos2 fungal histone deacetylase (HDAC) enzyme as well as affects the non-histone protein such as Hsp90. This formulation was developed by Mirati Therapeutics, Inc. and is under Phase II clinal trial against fungal infection. This drug is co-administrated with both azole and echinocandins, and is known for fungicidal activity against *Candida* sp. and *Aspergillus* sp. [119] (Table 2).

(f) Fosmanogepix (APX001)

Fosmanogepix is a glycosylphosphatidylinositol inhibitor developed by Amplyx Pharmaceuticals which is currently in Phase II clinical trials. It is metabolized into its active form, manogepix, which targets the enzyme Gwt1, responsible for the glycosylphosphatidylinositol (GPI) anchor biosynthesis [125,126]. Fosmanogepix was also reported to actively inhibit the growth of yeast, moulds, *Candida* sp., *Cryptococcus* sp., *Coccidioides* sp., and *Aspergillus* sp. [127] (Table 2) (Figure 9b).

**Table 2:** List of new antifungal drugs.

New Antifungal Agents Developed	Mode of Action	Developer	Activity Against	Clinical Trial Stage	Reference
VT-1598	Inhibits lanosterol 14α-demethylase	Mycovia Pharmaceuticals, Durham, NC, USA	Moulds, *Aspergillus* spp., *Rhizopus arrhizus*, and *Coccidioides*	Phase I	[102,120]
SUBA-itraconazole	Enhance the bioavailability of itraconazole by its solid dispersion in a pH-dependent matrix.	Mayne Pharma Ltd., Salisbury South, Australia	*Aspergillus* spp., *Blastomyces dermatitidis*	FDA-approved	[118]
Rezafungin	Inhibition of 1,3-β-D-glucan synthesis	Cidara Therapeutics, San Diego, CA, USA	*Candida* spp., *Aspergillus* spp., and *Pneumocystis* spp.	FDA-approved as an orphan drug for the treatment of vulvovaginal candidiasis	[119,121]
Ibrexafungin	Inhibition of 1,3-β-D-glucan synthesis	SCYNEXIS Inc., Jersey City, NJ, USA	*Candida* spp.	FDA-approved	[122]
Olorofim	Inhibition of dihydroorotate dehydrogenase responsible for pyrimidine biosynthesis.	Shionogi & Co., Ltd. And F2G Ltd., Osaka, Japan and Manchester, UK	*Aspergillus* and *Scedosporium* spp.	Phase II	[123]
Amphotericin B Cochleate (CAMB)	Cell wall disruption	Matinas BioPharma, Bedminster, NJ, USA.	*C. albicans*	Phase II	[124]
MGCD290	Inhibits Hos2 fungal histone deacetylase (HDAC) and also affects non-histone protein Hsp90	Mirati Therapeutics, Inc., San Diego, CA, USA	*Candida* and *Aspergillus* spp.	Phase II	[119]
Fosmanogepix (APX001)	Inhibition of glycosylphosphatidylinositol.	Amplyx Pharmaceuticals, San Diego, CA, USA	Yeast, moulds, *Candida*, *Cryptococcus*, *Coccidioides*, and *Aspergillus* spp.	Phase II	[125,126]
VL-2397	Unknown	Vical Pharmaceuticals, San Diego, CA, USA	*Aspergillus fumigatus.*	Phase II	[128,129]
T-2307	Fungal mitochondrial disruption	Toyama Chemical Co., Tokyo, Japan.	*Candida* spp. *Cryptococcus* and *Aspergillus* spp.	Phase I	[130]

(g) VL-2397

VL-2397, also known as ASP2397, was isolated from *Acremonium* sp. and formulated by Vical Pharmaceuticals. Fungi uptakes this drug through the siderophore iron transporter 1 (Sit1), leading to disruption of the intercellular process. It was reportedly effective against *Aspergillus fumigatus* under Phase II of clinical trials [121,128,129] (Table 2).

(h) T-2307

This arylamide compound developed by Toyama Chemical Co., Toyama, Japan, is in Phase I of clinical trials. The structure of this drug is similar to that of aromatic diamidines [131]. It mainly disrupts fungal mitochondrial membranes and is effective against *Candida* sp., *Cryptococcus* sp., and *Aspergillus* sp. [130,132] (Table 2) (Figure 9f).

(i) Retinoids and All-trans retinoic acid (ATRA)

Retinoids and all-trans retinoic acid (ATRA) are derived from Vitamin A. Studies have proved that these compounds have antifungal properties and can effectively treat various skin and systemic fungal infections. These compounds have shown a broad spectrum of activity against yeast, Candida albicans, and Aspergillus fumigatus [133,134]. Vitamin A serum is eligible for clinical practice to prevent skin and systemic fungal infection in psoriatic patients when treated with IL-17 inhibitor [135].

#### 3.3.2. Combination Therapy

Combination therapy is an effective approach to antifungal drug resistance and mainly helpful in extending the useful life of existing drugs. The combination of two drugs can be a much more effective way of killing the pathogen, reducing the pathogen population, and minimizing the chances of experiencing acquired resistance mutation [136,137]. Combination therapy also reduces the individual dosage of the drug, the length of treatment, and drug toxicity [136]. For instance, combination of amphotericin B and fluorocytosine is the best example of combination therapy for the treatment of persistent *Cryptococcal meningitis* and Candida fungal infections [138]. 

#### 3.3.3. Antifungal Stewardship

Antifungal stewardship refers to the method in which the diagnostics method and the usage and dosage of antifungal drugs are combined to obtain a gained better clinical outcome, which can reduce the emergence of resistance in fungi [139,140]. As antifungal management is mainly based on the guidelines used for the treatment and diagnostic tests that are safe for the patient, the combination of health professionals and public health centres following the guidelines for proper care of patients formed a stewardship team to execute the program [141,142]. However, several health centres lack antifungal stewardship due to unavailability or lack of access to long-term diagnostic tools, and, hence, failed to manage and execute the program. Thus, it is recommended for each and every health care centre and institution to conduct antifungal stewardship programs that contain guidelines and diagnostic tests to guide patients about the treatment duration, as well as guidance from the specialists and pharmacists to adopt the dosage and mode of administration of drugs [143]. The Mycoses Study Group Education and Research Consortium (MSGERC) has designed a set of core recommendations that can uplift the practice of antifungal stewardship. Their core recommendation includes integration of antifungal stewardship goals into hospital strategic management policies with the proper guidance of a senior leader and a core team. The core team should have the knowledge of and clinical expertise in invasive fungal infection management. The team should be able to develop collaborative strategies with the help of clinical specialists for better antifungal therapies. They also recommend proper diagnostic testing for Candida and Aspergillus species [140]. A study conducted by Valerio and team on antifungal stewardship in tertiary care institutes found that the annual antifungal drug expenditure was reduced within 2 years of implementation of an antifungal stewardship program. Along with that, the number of incidences was also reduced. This study helped to understand that the impact of antifungal stewardship can be an efficacious and cost-effective approach [144]. Considering all these factors, antifungal stewardship can significantly contribute to the prevention of drug resistance in fungi. 

#### 3.3.4. Potential Drug Targets to Overcome Antifungal Resistance

A better understanding of the drug resistance mechanism can assist in finding new drug targets which can be targeted for the designing and development of novel antifungal drugs. For instance, Hsp 90 of fungi are one major drug target, as they contribute to the cellular stress response in fungi [145]. However, incompetence in the Hsp90 function leads to resistance development in fungi against the azole and echinocandin classes [146]. Thus, inhibition of Hsp90, such as that exhibited by geldanamycin, was adopted to reduce resistance to caspofungin in *A. fumigatus* [147]. Additionally, calcineurin is another drug target which is required for growth, virulence, and drug resistance development in fungi such as *C. albicans*, *A. fumigatus*, and *C. neoformans* [148,149]. Hence, such essential enzymes of the fungi can be targeted for the designing and development of new antifungal agents to escape the drug resistance in pathogenic fungi. 

#### 3.3.5. One Health Approach in Combating Antifungal Resistance

The “One health” concept identifies that human, animals, and the environment are all indistinguishably connected and influential to one another [150]. This concept was first designed to combat the health of humans, plants, and animals during the emergence of the SARS-CoV and influenza virus in the year 2003–2004 and gained importance with the evolution of antimicrobial resistance in humans [151]. The number of patients who are highly susceptible to fungal infections is increasing as a result of changes in medical care, and this problem is made worse by the fact that our meagre supply of antifungal medications is being threatened (or compromised) by the emergence of drug-resistant fungi strains, which in some cases are linked to antifungal agents used in agriculture. The “One Health” idea seeks to educate scientists and decision-makers about these recognized and newly developing fungal dangers to global health by stressing the convergence of these dynamics. [150]. The American Academy of Microbiology organized a colloquium in October 2017 to address this issue, assembling a multidisciplinary and worldwide team of experts. This gathering took place ten years after the Academy hosted the first of its kind in 2007. One Health: Fungal Pathogens of Humans, Animals, and Plants, a new report, emphasizes the field’s extraordinary advancements and offers updated suggestions to deal with problems in public health and science in the modern world [150]. For instance, this concept has been used to closely analyse azole fungicide resistance in the environment and to reduce the burden of environmental resistance [152]. The “One Health” approach focuses on finding and prioritizing effective research methodologies that can be useful in developing various preventive guidelines and fill the major gaps in antifungal resistance conditions, as they can also originate from the environment, as well as from hospital facilities [8,153].

## 4. Conclusions

The rise in fungal infections has paradoxically advanced medicine. As a result, the frequent utilization of antifungal agents has also been transpired. In clinical setups, researchers and clinicians are facing the serious threat of antifungal resistance. Thus, along with the old strategies, some new methods are gaining popularity to tackle the growing drug resistance in fungi, as these new methods are more robust, and the accuracy lever is higher compared to the traditional ones. Additionally, new combinational therapies and drug trials are coming to light which will lead us closer to winning the war against antifungal resistance with better drugs with lower toxicity, fewer cross-interactions with side proteins, and higher specificity to the target sites. Ultimately, elucidating strategies to combat fungal pathogens in humans may cast light on how fungal infections that pose a worldwide danger to biodiversity can be defeated. 

## Data Availability

Not applicable.

## References

[1] Kainz K., Bauer M.A., Madeo F., Carmona-Gutierrez D. (2020). Fungal Infections in Humans: The Silent Crisis. Microb. Cell.

[2] Papon N., Bougnoux M.-E., d’Enfert C. (2020). Tracing the Origin of Invasive Fungal Infections. Trends Microbiol..

[3] Firacative C. (2020). Invasive Fungal Disease in Humans: Are We Aware of the Real Impact?. Memórias Do Inst. Oswaldo Cruz..

[4] WHO Fungal Priority Pathogens List to Guide Research, Development and Public Health Action. https://www.who.int/publications-detail-redirect/9789240060241.

[5] Brown G.D., Denning D.W., Gow N.A.R., Levitz S.M., Netea M.G., White T.C. (2012). Hidden Killers: Human Fungal Infections. Sci. Transl. Med..

[6] Bongomin F., Gago S., Oladele R.O., Denning D.W. (2017). Global and Multi-National Prevalence of Fungal Diseases—Estimate Precision. J. Fungi.

[7] Prakash H., Chakrabarti A. (2019). Global Epidemiology of Mucormycosis. J. Fungi.

[8] Verweij P.E., Lucas J.A., Arendrup M.C., Bowyer P., Brinkmann A.J.F., Denning D.W., Dyer P.S., Fisher M.C., Geenen P.L., Gisi U. (2020). The One Health Problem of Azole Resistance in Aspergillus Fumigatus: Current Insights and Future Research Agenda. Fungal Biol. Rev..

[9] Rhodes J., Fisher M.C. (2019). Global Epidemiology of Emerging Candida Auris. Curr. Opin. Microbiol..

[10] Bilal H., Hou B., Shafiq M., Chen X., Shahid M.A., Zeng Y. (2022). Antifungal Susceptibility Pattern of Candida Isolated from Cutaneous Candidiasis Patients in Eastern Guangdong Region: A Retrospective Study of the Past 10 Years. Front. Microbiol..

[11] Bilal H., Shafiq M., Hou B., Islam R., Khan M.N., Khan R.U., Zeng Y. (2022). Distribution and Antifungal Susceptibility Pattern of Candida Species from Mainland China: A Systematic Analysis. Virulence.

[12] Janbon G., Quintin J., Lanternier F., d’Enfert C. (2019). Studying Fungal Pathogens of Humans and Fungal Infections: Fungal Diversity and Diversity of Approaches. Microbes Infect..

[13] Enoch D.A., Yang H., Aliyu S.H., Micallef C., Lion T. (2017). The Changing Epidemiology of Invasive Fungal Infections. Human Fungal Pathogen Identification: Methods and Protocols.

[14] Song G., Liang G., Liu W. (2020). Fungal Co-Infections Associated with Global COVID-19 Pandemic: A Clinical and Diagnostic Perspective from China. Mycopathologia.

[15] Pushparaj K., Kuchi Bhotla H., Arumugam V.A., Pappusamy M., Easwaran M., Liu W.-C., Issara U., Rengasamy K.R.R., Meyyazhagan A., Balasubramanian B. (2022). Mucormycosis (Black Fungus) Ensuing COVID-19 and Comorbidity Meets-Magnifying Global Pandemic Grieve and Catastrophe Begins. Sci. Total Environ..

[16] Drissi C. (2021). Black Fungus, the Darker Side of COVID-19. J. Neuroradiol..

[17] Shapiro R.S., Robbins N., Cowen L.E. (2011). Regulatory Circuitry Governing Fungal Development, Drug Resistance, and Disease. Microbiol. Mol. Biol. Rev..

[18] Perlin D.S., Rautemaa-Richardson R., Alastruey-Izquierdo A. (2017). The Global Problem of Antifungal Resistance: Prevalence, Mechanisms, and Management. Lancet Infect. Dis..

[19] Robbins N., Caplan T., Cowen L.E. (2017). Molecular Evolution of Antifungal Drug Resistance. Annu. Rev. Microbiol..

[20] Edlind Thomas D., Katiyar Santosh K. (2010). Mutational Analysis of Flucytosine Resistance in Candida Glabrata. Antimicrob. Agents Chemother..

[21] Berman J., Krysan D.J. (2020). Drug Resistance and Tolerance in Fungi. Nat. Rev. Microbiol..

[22] Shor E., Perlin D.S. (2015). Coping with Stress and the Emergence of Multidrug Resistance in Fungi. PLoS Pathog..

[23] Meletiadis J., Antachopoulos C., Stergiopoulou T., Pournaras S., Roilides E., Walsh T.J. (2007). Differential Fungicidal Activities of Amphotericin B and Voriconazole against Aspergillus Species Determined by Microbroth Methodology. Antimicrob. Agents Chemother..

[24] Geißel B., Loiko V., Klugherz I., Zhu Z., Wagener N., Kurzai O., van den Hondel C.A.M.J.J., Wagener J. (2018). Azole-Induced Cell Wall Carbohydrate Patches Kill Aspergillus Fumigatus. Nat. Commun..

[25] Patil A., Majumdar S. (2017). Echinocandins in Antifungal Pharmacotherapy. J. Pharm. Pharmacol..

[26] Carmona E.M., Limper A.H. (2017). Overview of Treatment Approaches for Fungal Infections. Clin. Chest Med..

[27] Fisher M.C., Hawkins N.J., Sanglard D., Gurr S.J. (2018). Worldwide Emergence of Resistance to Antifungal Drugs Challenges Human Health and Food Security. Science.

[28] Fisher M.C., Alastruey-Izquierdo A., Berman J., Bicanic T., Bignell E.M., Bowyer P., Bromley M., Brüggemann R., Garber G., Cornely O.A. (2022). Tackling the Emerging Threat of Antifungal Resistance to Human Health. Nat. Rev. Microbiol..

[29] Revie N.M., Iyer K.R., Robbins N., Cowen L.E. (2018). Antifungal Drug Resistance: Evolution, Mechanisms and Impact. Curr. Opin. Microbiol..

[30] Zheng Y.-H., Ma Y.-Y., Ding Y., Chen X.-Q., Gao G.-X. (2018). An Insight into New Strategies to Combat Antifungal Drug Resistance. Drug Des. Dev..

[31] Pathadka S., Yan V.K.C., Neoh C.F., Al-Badriyeh D., Kong D.C.M., Slavin M.A., Cowling B.J., Hung I.F.N., Wong I.C.K., Chan E.W. (2022). Global Consumption Trend of Antifungal Agents in Humans From 2008 to 2018: Data From 65 Middle- and High-Income Countries. Drugs.

[32] Hoenigl M., Sprute R., Egger M., Arastehfar A., Cornely O.A., Krause R., Lass-Flörl C., Prattes J., Spec A., Thompson G.R. (2021). The Antifungal Pipeline: Fosmanogepix, Ibrexafungerp, Olorofim, Opelconazole, and Rezafungin. Drugs.

[33] Perfect J.R. (2017). The Antifungal Pipeline: A Reality Check. Nat. Rev. Drug Discov..

[34] Ostrosky-Zeichner L., Casadevall A., Galgiani J.N., Odds F.C., Rex J.H. (2010). An Insight into the Antifungal Pipeline: Selected New Molecules and Beyond. Nat. Rev. Drug Discov..

[35] Campoy S., Adrio J.L. (2017). Antifungals. Biochem. Pharmacol..

[36] Odds F.C., Brown A.J.P., Gow N.A.R. (2003). Antifungal Agents: Mechanisms of Action. Trends Microbiol..

[37] Zotchev S.B. (2003). Polyene Macrolide Antibiotics and Their Applications in Human Therapy. Curr. Med. Chem..

[38] Hamilton-Miller J.M. (1973). Chemistry and Biology of the Polyene Macrolide Antibiotics. Bacteriol. Rev..

[39] Kinsky S.C., Gottlieb D., Shaw P.D. (1967). Polyene Antibiotics. Antibiotics: Volume I Mechanism of Action.

[40] Bekersky I., Fielding R.M., Dressler D.E., Lee J.W., Buell D.N., Walsh T.J. (2002). Pharmacokinetics, Excretion, and Mass Balance of Liposomal Amphotericin B (AmBisome) and Amphotericin B Deoxycholate in Humans. Antimicrob. Agents Chemother..

[41] Mesa-Arango A.C., Scorzoni L., Zaragoza O. (2012). It Only Takes One to Do Many Jobs: Amphotericin B as Antifungal and Immunomodulatory Drug. Front. Microbiol..

[42] Kristanc L., Božič B., Jokhadar Š.Z., Dolenc M.S., Gomišček G. (2019). The Pore-Forming Action of Polyenes: From Model Membranes to Living Organisms. Biochim. Et Biophys. Acta (BBA)-Biomembr..

[43] Quezada H., Martínez-Vázquez M., López-Jácome E., González-Pedrajo B., Andrade Á., Fernández-Presas A.M., Tovar-García A., García-Contreras R. (2020). Repurposed Anti-Cancer Drugs: The Future for Anti-Infective Therapy?. Expert Rev. Anti Infect..

[44] Rojas E., Herrera L.A., Sordo M., Gonsebatt M.E., Montero R., Rodríguez R., Ostrosky-Wegman P. (1993). Mitotic Index and Cell Proliferation Kinetics for Identification of Antineoplastic Activity. Anticancer Drugs.

[45] Longley D.B., Harkin D.P., Johnston P.G. (2003). 5-Fluorouracil: Mechanisms of Action and Clinical Strategies. Nat. Rev. Cancer.

[46] Moudi M., Go R., Yien C.Y.S., Nazre M. (2013). Vinca Alkaloids. Int. J. Prev. Med..

[47] Weaver B.A. (2014). How Taxol/Paclitaxel Kills Cancer Cells. Mol. Biol. Cell.

[48] Perfect J.R., Dismukes W.E., Dromer F., Goldman D.L., Graybill J.R., Hamill R.J., Harrison T.S., Larsen R.A., Lortholary O., Nguyen M.-H. (2010). Clinical Practice Guidelines for the Management of Cryptococcal Disease: 2010 Update by the Infectious Diseases Society of America. Clin. Infect. Dis..

[49] Cornely O.A., Bassetti M., Calandra T., Garbino J., Kullberg B.J., Lortholary O., Meersseman W., Akova M., Arendrup M.C., Arikan-Akdagli S. (2012). ESCMID* Guideline for the Diagnosis and Management of Candida Diseases 2012: Non-Neutropenic Adult Patients. Clin. Microbiol. Infect..

[50] Tobias J.S., Wrigley P.F., Shaw E. (1976). Combination Antifungal Therapy for Cryptococcal Meningitis. Postgrad. Med. J..

[51] Vermes A., Guchelaar H.-J., Dankert J. (2000). Flucytosine: A Review of Its Pharmacology, Clinical Indications, Pharmacokinetics, Toxicity and Drug Interactions. J. Antimicrob. Chemother..

[52] Heidemann H.T., Brune K.H., Sabra R., Branch R.A. (1992). Acute and Chronic Effects of Flucytosine on Amphotericin B Nephrotoxicity in Rats. Antimicrob. Agents Chemother..

[53] Schwarz P., Janbon G., Dromer F., Lortholary O., Dannaoui E. (2007). Combination of Amphotericin B with Flucytosine Is Active In Vitro against Flucytosine-Resistant Isolates of Cryptococcus Neoformans. Antimicrob. Agents Chemother..

[54] Houšť J., Spížek J., Havlíček V. (2020). Antifungal Drugs. Metabolites.

[55] Padda I.S., Parmar M. (2022). Flucytosine. StatPearls.

[56] Gsaller F., Furukawa T., Carr P.D., Rash B., Jöchl C., Bertuzzi M., Bignell E.M., Bromley M.J. (2018). Mechanistic Basis of PH-Dependent 5-Flucytosine Resistance in Aspergillus Fumigatus. Antimicrob. Agents Chemother..

[57] García-García I., Borobia A.M. (2021). Current Approaches and Future Strategies for the Implementation of Pharmacogenomics in the Clinical Use of Azole Antifungal Drugs. Expert Opin. Drug Metab. Toxicol..

[58] Howard K.C., Dennis E.K., Watt D.S., Garneau-Tsodikova S. (2020). A Comprehensive Overview of the Medicinal Chemistry of Antifungal Drugs: Perspectives and Promise. Chem. Soc. Rev..

[59] Brand S.R., Degenhardt T.P., Person K., Sobel J.D., Nyirjesy P., Schotzinger R.J., Tavakkol A. (2018). A Phase 2, Randomized, Double-Blind, Placebo-Controlled, Dose-Ranging Study to Evaluate the Efficacy and Safety of Orally Administered VT-1161 in the Treatment of Recurrent Vulvovaginal Candidiasis. Am. J. Obs. Gynecol..

[60] Cui X., Wang L., Lü Y., Yue C. (2022). Development and Research Progress of Anti-Drug Resistant Fungal Drugs. J. Infect. Public Health.

[61] Neochoritis C.G., Zhao T., Dömling A. (2019). Tetrazoles via Multicomponent Reactions. Chem. Rev..

[62] Monk Brian C., Keniya Mikhail V., Manya S., Wilson Rajni K., Graham Danyon O., Hassan Harith F., Danni C., Tyndall Joel D.A. (2018). Azole Resistance Reduces Susceptibility to the Tetrazole Antifungal VT-1161. Antimicrob. Agents Chemother..

[63] Hoekstra W.J., Garvey E.P., Moore W.R., Rafferty S.W., Yates C.M., Schotzinger R.J. (2014). Design and Optimization of Highly-Selective Fungal CYP51 Inhibitors. Bioorganic Med. Chem. Lett..

[64] Warrilow A.G.S., Hull C.M., Parker J.E., Garvey E.P., Hoekstra W.J., Moore W.R., Schotzinger R.J., Kelly D.E., Kelly S.L. (2014). The Clinical Candidate VT-1161 Is a Highly Potent Inhibitor of Candida Albicans CYP51 but Fails To Bind the Human Enzyme. Antimicrob. Agents Chemother..

[65] Wiederhold N.P., Najvar L.K., Garvey E.P., Brand S.R., Xu X., Ottinger E.A., Alimardanov A., Cradock J., Behnke M., Hoekstra W.J. (2018). The Fungal Cyp51 Inhibitor VT-1129 Is Efficacious in an Experimental Model of Cryptococcal Meningitis. Antimicrob. Agents Chemother..

[66] Sobel J.D., Nyirjesy P. (2021). Oteseconazole: An Advance in Treatment of Recurrent Vulvovaginal Candidiasis. Future Microbiol..

[67] Lockhart Shawn R., Fothergill Annette W., Naureen I., Bolden Carol B., Grossman Nina T., Garvey Edward P., Brand Stephen R., Hoekstra William J., Schotzinger Robert J., Elizabeth O. (2016). The Investigational Fungal Cyp51 Inhibitor VT-1129 Demonstrates Potent In Vitro Activity against Cryptococcus Neoformans and Cryptococcus Gattii. Antimicrob. Agents Chemother..

[68] Kathiravan M.K., Salake A.B., Chothe A.S., Dudhe P.B., Watode R.P., Mukta M.S., Gadhwe S. (2012). The Biology and Chemistry of Antifungal Agents: A Review. Bioorg. Med. Chem..

[69] Cadena J., Thompson G.R., Patterson T.F. (2016). Invasive Aspergillosis: Current Strategies for Diagnosis and Management. Infect. Dis. Clin..

[70] Zhou C.-H., Wang Y. (2012). Recent Researches in Triazole Compounds as Medicinal Drugs. Curr. Med. Chem..

[71] Chang Y.-L., Yu S.-J., Heitman J., Wellington M., Chen Y.-L. (2017). New Facets of Antifungal Therapy. Virulence.

[72] Lass-Flörl C. (2011). Triazole Antifungal Agents in Invasive Fungal Infections. Drugs.

[73] Kernt M., Kampik A. (2010). Endophthalmitis: Pathogenesis, Clinical Presentation, Management, and Perspectives. Clin. Ophthalmol..

[74] Kofla G., Ruhnke M. (2011). Pharmacology and Metabolism of Anidulafungin, Caspofungin and Micafungin in the Treatment of Invasive Candidosis—Review of the Literature. Eur. J. Med. Res..

[75] Stover K.R., Farley J.M., Kyle P.B., Cleary J.D. (2014). Cardiac Toxicity of Some Echinocandin Antifungals. Expert Opin. Drug Saf..

[76] Denning D.W. (2002). Echinocandins: A New Class of Antifungal. J. Antimicrob. Chemother..

[77] Bachmann S.P., Patterson T.F., López-Ribot J.L. (2002). In Vitro Activity of Caspofungin (MK-0991) against Candida Albicans Clinical Isolates Displaying Different Mechanisms of Azole Resistance. J. Clin. Microbiol..

[78] Gil-Lamaignere C., Salvenmoser S., Hess R., Müller F.-M.C. (2004). Micafungin Enhances Neutrophil Fungicidal Functions against Candida Pseudohyphae. Antimicrob. Agents Chemother..

[79] Pontón J. (2008). [The fungal cell wall and the mechanism of action of anidulafungin]. Rev. Iberoam. Micol..

[80] Felton T., Troke P.F., Hope W.W. (2014). Tissue Penetration of Antifungal Agents. Clin. Microbiol. Rev..

[81] Sucher A.J., Chahine E.B., Balcer H.E. (2009). Echinocandins: The Newest Class of Antifungals. Ann. Pharm..

[82] Chandrasekar P.H., Sobel J.D. (2006). Micafungin: A New Echinocandin. Clin. Infect. Dis..

[83] Bowman J.C., Hicks P.S., Kurtz M.B., Rosen H., Schmatz D.M., Liberator P.A., Douglas C.M. (2002). The Antifungal Echinocandin Caspofungin Acetate Kills Growing Cells of Aspergillus Fumigatus in Vitro. Antimicrob. Agents Chemother..

[84] Kurtz M.B., Heath I.B., Marrinan J., Dreikorn S., Onishi J., Douglas C. (1994). Morphological Effects of Lipopeptides against Aspergillus Fumigatus Correlate with Activities against (1,3)-Beta-D-Glucan Synthase. Antimicrob. Agents Chemother..

[85] Sanglard D. (2002). Resistance of Human Fungal Pathogens to Antifungal Drugs. Curr. Opin. Microbiol..

[86] Hull C.M., Bader O., Parker J.E., Weig M., Gross U., Warrilo A.G.S., Kelly D.E., Kelly S.L. (2012). Two Clinical Isolates of Candida Glabrata Exhibiting Reduced Sensitivity to Amphotericin B Both Harbor Mutations in ERG2. Antimicrob. Agents Chemother..

[87] Carolus H., Pierson S., Muñoz J.F., Subotić A., Cruz R.B., Cuomo C.A., Van Dijck P. (2021). Genome-Wide Analysis of Experimentally Evolved Candida Auris Reveals Multiple Novel Mechanisms of Multidrug Resistance. mBio.

[88] Blatzer M., Blum G., Jukic E., Posch W., Gruber P., Nagl M., Binder U., Maurer E., Sarg B., Lindner H. (2015). Blocking Hsp70 Enhances the Efficiency of Amphotericin B Treatment against Resistant Aspergillus Terreus Strains. Antimicrob. Agents Chemother..

[89] Posch W., Blatzer M., Wilflingseder D., Lass-Flörl C. (2018). Aspergillus Terreus: Novel Lessons Learned on Amphotericin B Resistance. Med. Mycol..

[90] Carolus H., Pierson S., Lagrou K., Van Dijck P. (2020). Amphotericin B and Other Polyenes—Discovery, Clinical Use, Mode of Action and Drug Resistance. J. Fungi.

[91] Delma F.Z., Al-Hatmi A.M.S., Brüggemann R.J.M., Melchers W.J.G., de Hoog S., Verweij P.E., Buil J.B. (2021). Molecular Mechanisms of 5-Fluorocytosine Resistance in Yeasts and Filamentous Fungi. J. Fungi.

[92] Papon N., Noël T., Florent M., Gibot-Leclerc S., Jean D., Chastin C., Villard J., Chapeland-Leclerc F. (2007). Molecular Mechanism of Flucytosine Resistance in Candida Lusitaniae: Contribution of the FCY2, FCY1, and FUR1 Genes to 5-Fluorouracil and Fluconazole Cross-Resistance. Antimicrob. Agents Chemother..

[93] Burks C., Darby A., Gómez Londoño L., Momany M., Brewer M.T. (2021). Azole-Resistant Aspergillus Fumigatus in the Environment: Identifying Key Reservoirs and Hotspots of Antifungal Resistance. PLoS Pathog..

[94] Sharma C., Chowdhary A. (2017). Molecular Bases of Antifungal Resistance in Filamentous Fungi. Int. J. Antimicrob. Agents.

[95] Cowen L.E., Sanglard D., Howard S.J., Rogers P.D., Perlin D.S. (2014). Mechanisms of Antifungal Drug Resistance. Cold Spring Harb. Perspect. Med..

[96] Whaley S.G., Rogers P.D. (2016). Azole Resistance in Candida Glabrata. Curr. Infect. Dis. Rep..

[97] Pinjon E., Moran G.P., Coleman D.C., Sullivan D.J. (2005). Azole Susceptibility and Resistance in Candida Dubliniensis. Biochem. Soc. Trans..

[98] Whaley S.G., Berkow E.L., Rybak J.M., Nishimoto A.T., Barker K.S., Rogers P.D. (2017). Azole Antifungal Resistance in Candida Albicans and Emerging Non-Albicans Candida Species. Front. Microbiol..

[99] Pfaller M.A., Diekema D.J., Turnidge J.D., Castanheira M., Jones R.N. (2019). Twenty Years of the SENTRY Antifungal Surveillance Program: Results for Candida Species From 1997–2016. Open Forum Infect. Dis..

[100] Rodrigues C.F., Rodrigues M.E., Henriques M. (2018). Susceptibility of Candida Glabrata Biofilms to Echinocandins: Alterations in the Matrix Composition. Biofouling.

[101] Liu C., Shi C., Mao F., Xu Y., Liu J., Wei B., Zhu J., Xiang M., Li J. (2014). Discovery of New Imidazole Derivatives Containing the 2,4-Dienone Motif with Broad-Spectrum Antifungal and Antibacterial Activity. Molecules.

[102] Nishimoto Andrew T., Wiederhold Nathan P., Flowers Stephanie A., Zhang Q., Kelly Steven L., Joachim M., Yates Christopher M., Hoekstra William J., Schotzinger Robert J., Garvey Edward P. (2019). In Vitro Activities of the Novel Investigational Tetrazoles VT-1161 and VT-1598 Compared to the Triazole Antifungals against Azole-Resistant Strains and Clinical Isolates of Candida Albicans. Antimicrob. Agents Chemother..

[103] Desai J.V., Mitchell A.P., Andes D.R. (2014). Fungal Biofilms, Drug Resistance, and Recurrent Infection. Cold Spring Harb. Perspect. Med..

[104] Gebreyohannes G., Nyerere A., Bii C., Sbhatu D.B. (2019). Challenges of Intervention, Treatment, and Antibiotic Resistance of Biofilm-Forming Microorganisms. Heliyon.

[105] Rajendran R., Sherry L., Deshpande A., Johnson E.M., Hanson M.F., Williams C., Munro C.A., Jones B.L., Ramage G. (2016). A Prospective Surveillance Study of Candidaemia: Epidemiology, Risk Factors, Antifungal Treatment and Outcome in Hospitalized Patients. Front. Microbiol..

[106] Rajendran R., Sherry L., Nile C.J., Sherriff A., Johnson E.M., Hanson M.F., Williams C., Munro C.A., Jones B.J., Ramage G. (2016). Biofilm Formation Is a Risk Factor for Mortality in Patients with Candida Albicans Bloodstream Infection-Scotland, 2012–2013. Clin. Microbiol. Infect..

[107] Sherry L., Ramage G., Kean R., Borman A., Johnson E.M., Richardson M.D., Rautemaa-Richardson R. (2017). Biofilm-Forming Capability of Highly Virulent, Multidrug-Resistant Candida Auris. Emerg. Infect. Dis..

[108] Ramage G., Rajendran R., Sherry L., Williams C. (2012). Fungal Biofilm Resistance. Int. J. Microbiol..

[109] Perlin D.S., Shor E., Zhao Y. (2015). Update on Antifungal Drug Resistance. Curr. Clin. Microbiol. Rep..

[110] Niimi K., Maki K., Ikeda F., Holmes A.R., Lamping E., Niimi M., Monk B.C., Cannon R.D. (2006). Overexpression of Candida Albicans CDR1, CDR2, or MDR1 Does Not Produce Significant Changes in Echinocandin Susceptibility. Antimicrob. Agents Chemother..

[111] Garcia-Effron G., Lee S., Park S., Cleary J.D., Perlin D.S. (2009). Effect of Candida Glabrata FKS1 and FKS2 Mutations on Echinocandin Sensitivity and Kinetics of 1,3-β-d-Glucan Synthase: Implication for the Existing Susceptibility Breakpoint. Antimicrob. Agents Chemother..

[112] Perlin D.S. (2011). Current Perspectives on Echinocandin Class Drugs. Future Microbiol..

[113] Morio F., Loge C., Besse B., Hennequin C., Le Pape P. (2010). Screening for Amino Acid Substitutions in the Candida Albicans Erg11 Protein of Azole-Susceptible and Azole-Resistant Clinical Isolates: New Substitutions and a Review of the Literature. Diagn. Microbiol. Infect. Dis..

[114] Sanglard D., Coste A.T. (2015). Activity of Isavuconazole and Other Azoles against Candida Clinical Isolates and Yeast Model Systems with Known Azole Resistance Mechanisms. Antimicrob. Agents Chemother..

[115] Prasad R., Banerjee A., Shah A.H. (2017). Resistance to Antifungal Therapies. Essays Biochem..

[116] Holmes A.R., Cardno T.S., Strouse J.J., Ivnitski-Steele I., Keniya M.V., Lackovic K., Monk B.C., Sklar L.A., Cannon R.D. (2016). Targeting Efflux Pumps to Overcome Antifungal Drug Resistance. Future Med. Chem..

[117] Chang W., Liu J., Zhang M., Shi H., Zheng S., Jin X., Gao Y., Wang S., Ji A., Lou H. (2018). Efflux Pump-Mediated Resistance to Antifungal Compounds Can Be Prevented by Conjugation with Triphenylphosphonium Cation. Nat. Commun..

[118] Abbotsford J., Foley D.A., Goff Z., Bowen A.C., Blyth C.C., Yeoh D.K. (2021). Clinical Experience with SUBA-Itraconazole at a Tertiary Paediatric Hospital. J. Antimicrob. Chemother..

[119] Gintjee T.J., Donnelley M.A., Thompson G.R. (2020). Aspiring Antifungals: Review of Current Antifungal Pipeline Developments. J. Fungi.

[120] Hargrove T.Y., Garvey E.P., Hoekstra W.J., Yates C.M., Wawrzak Z., Rachakonda G., Villalta F., Lepesheva G.I. (2017). Crystal Structure of the New Investigational Drug Candidate VT-1598 in Complex with Aspergillus Fumigatus Sterol 14α-Demethylase Provides Insights into Its Broad-Spectrum Antifungal Activity. Antimicrob. Agents Chemother..

[121] Van Daele R., Spriet I., Wauters J., Maertens J., Mercier T., Van Hecke S., Brüggemann R. (2019). Antifungal Drugs: What Brings the Future?. Med. Mycol..

[122] Davis M.R., Donnelley M.A., Thompson G.R. (2020). Ibrexafungerp: A Novel Oral Glucan Synthase Inhibitor. Med. Mycol..

[123] Rivero-Menendez O., Cuenca-Estrella M., Alastruey-Izquierdo A. (2019). In Vitro Activity of Olorofim (F901318) against Clinical Isolates of Cryptic Species of Aspergillus by EUCAST and CLSI Methodologies. J. Antimicrob. Chemother..

[124] Santangelo R., Paderu P., Delmas G., Chen Z.-W., Mannino R., Zarif L., Perlin D.S. (2000). Efficacy of Oral Cochleate-Amphotericin B in a Mouse Model of Systemic Candidiasis. Antimicrob. Agents Chemother..

[125] Alkhazraji S., Gebremariam T., Alqarihi A., Gu Y., Mamouei Z., Singh S., Wiederhold N.P., Shaw K.J., Ibrahim A.S. (2020). Fosmanogepix (APX001) Is Effective in the Treatment of Immunocompromised Mice Infected with Invasive Pulmonary Scedosporiosis or Disseminated Fusariosis. Antimicrob. Agents Chemother..

[126] Shaw K.J., Ibrahim A.S. (2020). Fosmanogepix: A Review of the First-in-Class Broad Spectrum Agent for the Treatment of Invasive Fungal Infections. J. Fungi.

[127] McCarthy M.W., Kontoyiannis D.P., Cornely O.A., Perfect J.R., Walsh T.J. (2017). Novel Agents and Drug Targets to Meet the Challenges of Resistant Fungi. J. Infect. Dis..

[128] Kovanda Laura L., Sullivan Sean M., Smith Larry R., Desai Amit V., Bonate Pete L., Hope William W. (2019). Population Pharmacokinetic Modeling of VL-2397, a Novel Systemic Antifungal Agent: Analysis of a Single- and Multiple-Ascending-Dose Study in Healthy Subjects. Antimicrob. Agents Chemother..

[129] Anna-Maria D., Matthias M., Aguiar Mario M., Vasyl I., David T., Joachim P., Clemens D., Martin H., Sullivan Sean M., Smith Larry R. (2019). The Siderophore Transporter Sit1 Determines Susceptibility to the Antifungal VL-2397. Antimicrob. Agents Chemother..

[130] Nishikawa H., Yamada E., Shibata T., Uchihashi S., Fan H., Hayakawa H., Nomura N., Mitsuyama J. (2010). Uptake of T-2307, a Novel Arylamidine, in Candida Albicans. J. Antimicrob. Chemother..

[131] Mitsuyama J., Nomura N., Hashimoto K., Yamada E., Nishikawa H., K M., Kimura A., Todo Y., Narita H. (2008). In Vitro and In Vivo Antifungal Activities of T-2307, a Novel Arylamidine. Antimicrob. Agents Chemother..

[132] Wiederhold N.P. (2021). Review of T-2307, an Investigational Agent That Causes Collapse of Fungal Mitochondrial Membrane Potential. J. Fungi.

[133] Campione E., Gaziano R., Marino D., Orlandi A. (2016). Fungistatic Activity of All-Trans Retinoic Acid against Aspergillus Fumigatus and Candida Albicans. Drug Des. Dev..

[134] Cosio T., Gaziano R., Zuccari G., Costanza G., Grelli S., Di Francesco P., Bianchi L., Campione E. (2021). Retinoids in Fungal Infections: From Bench to Bedside. Pharmaceuticals.

[135] Campione E., Cosio T., Lanna C., Mazzilli S., Ventura A., Dika E., Gaziano R., Dattola A., Candi E., Bianchi L. (2020). Predictive Role of Vitamin A Serum Concentration in Psoriatic Patients Treated with IL-17 Inhibitors to Prevent Skin and Systemic Fungal Infections. J. Pharmacol. Sci..

[136] Hill J.A., Cowen L.E. (2015). Using Combination Therapy to Thwart Drug Resistance. Future Microbiol..

[137] Livengood S.J., Drew R.H., Perfect J.R. (2020). Combination Therapy for Invasive Fungal Infections. Curr. Fungal Infect. Rep..

[138] Evans E.G.V. (2001). The Rationale for Combination Therapy. Br. J. Dermatol..

[139] Ruhnke M. (2014). Antifungal Stewardship in Invasive Candida Infections. Clin. Microbiol. Infect..

[140] Johnson M.D., Lewis R.E., Dodds Ashley E.S., Ostrosky-Zeichner L., Zaoutis T., Thompson G.R., Andes D.R., Walsh T.J., Pappas P.G., Cornely O.A. (2020). Core Recommendations for Antifungal Stewardship: A Statement of the Mycoses Study Group Education and Research Consortium. J. Infect. Dis..

[141] Valerio M., Muñoz P., Rodríguez-González C., Sanjurjo M., Guinea J., Bouza E. (2015). Training Should Be the First Step toward an Antifungal Stewardship Program. Enferm. Infecc. Y Microbiol. Clínica.

[142] Urbancic K.F., Thursky K., Kong D.C.M., Johnson P.D.R., Slavin M.A. (2018). Antifungal Stewardship: Developments in the Field. Curr. Opin. Infect. Dis..

[143] Micallef C., Aliyu S.H., Santos R., Brown N.M., Rosembert D., Enoch D.A. (2015). Introduction of an Antifungal Stewardship Programme Targeting High-Cost Antifungals at a Tertiary Hospital in Cambridge, England. J. Antimicrob. Chemother..

[144] Valerio M., Muñoz P., Rodríguez C.G., Caliz B., Padilla B., Fernández-Cruz A., Sánchez-Somolinos M., Gijón P., Peral J., Gayoso J. (2015). Antifungal Stewardship in a Tertiary-Care Institution: A Bedside Intervention. Clin. Microbiol. Infect..

[145] Leach M.D., Klipp E., Cowen L.E., Brown A.J.P. (2012). Fungal Hsp90: A Biological Transistor That Tunes Cellular Outputs to Thermal Inputs. Nat. Rev. Microbiol..

[146] Singh S.D., Robbins N., Zaas A.K., Schell W.A., Perfect J.R., Cowen L.E. (2009). Hsp90 Governs Echinocandin Resistance in the Pathogenic Yeast Candida Albicans via Calcineurin. PLoS Pathog..

[147] Lamoth F., Juvvadi P.R., Gehrke C., Steinbach W.J. (2013). In Vitro Activity of Calcineurin and Heat Shock Protein 90 Inhibitors against Aspergillus Fumigatus Azole- and Echinocandin-Resistant Strains. Antimicrob. Agents Chemother..

[148] Chen Y.-L., Lehman V.N., Lewit Y., Averette A.F., Heitman J. (2013). Calcineurin Governs Thermotolerance and Virulence of Cryptococcus Gattii. G3 (Bethesda).

[149] Chen Y.-L., Brand A., Morrison E.L., Silao F.G.S., Bigol U.G., Malbas F.F., Nett J.E., Andes D.R., Solis N.V., Filler S.G. (2011). Calcineurin Controls Drug Tolerance, Hyphal Growth, and Virulence in Candida Dubliniensis. Eukaryot. Cell.

[150] (2019). One Health: Fungal Pathogens of Humans, Animals, and Plants: Report on an American Academy of Microbiology Colloquium Held in Washington, DC, on 18 October 2017.

[151] Schneider M.C., Munoz-Zanzi C., Min K., Aldighieri S. (2018). “One Health” From Concept to Application in the Global World; Oxford Research Encyclopedia, Global Public Health.

[152] Chowdhary A., Meis J. (2018). Emergence of Azole Resistant Aspergillus Fumigatus and One Health: Time to Implement Environmental Stewardship. Environ. Microbiol..

[153] Banerjee S., Denning D.W., Chakrabarti A. (2021). One Health Aspects & Priority Roadmap for Fungal Diseases: A Mini-Review. Indian J. Med. Res..

